# Five new species of *Drosophilaguarani* group from the Andes of southern Ecuador (Diptera, Drosophilidae)

**DOI:** 10.3897/zookeys.781.22841

**Published:** 2018-08-15

**Authors:** Ana Danitza Peñafiel-Vinueza, Violeta Rafael

**Affiliations:** 1 Laboratorio de Genética Evolutiva, Facultad de Ciencias Exactas y Naturales, Pontificia Universidad Católica del Ecuador, Av. 12 de Octubre y Roca, Aptdo. 17-01-2184, Quito, Ecuador Pontificia Universidad Católica del Ecuador Quito Ecuador

**Keywords:** *
Drosophila
*, *guarani* group, new species, southern Ecuador, terminalia

## Abstract

Five species of the genus *Drosophila* are described and illustrated: *D.zamorana***sp. n.**, *D.quinarensis***sp. n.**, *D.sachapuyu***sp. n.**, *D.caxarumi***sp. n.**, and *D.misi***sp. n.** from the cloud forests of the Podocarpus National Park, in the southern Ecuadorian Andes. Flies were captured using plastic bottles containing pieces of fermented banana with yeast. All the species were found to belong to the *Drosophilaguarani* species-group.

## Introduction

The *guarani* group of *Drosophila* was proposed by Dobzhansky & Pavan (1943) and included 12 species until 1993: *D.alexandrei* Cordeiro, 1951; *D.araucana* Brncic, 1957; *D.griseolineata* Duda, 1927; *D.guaraja* King, 1947; *D.guaru* Dobzhansky & Pavan, 1943; *D.huilliche* Brncic, 1957; *D.limbinervis* Duda, 1925; *D.maculifrons* Duda, 1927; *D.ornatifrons* Duda, 1927; *D.peruensis* Wheeler, 1959; *D.subbadia* Patterson & Mainland, 1943, and *D.tucumana* Vilela & Pereira, 1985 (Vilela and Pereira 1993). There was a misidentification of *D.peruensis* which would have been the thirteen member of the *guarani* group, *Drosophilaurubamba* (Vilela & Pereira, 1993). Ratcov and Vilela 2007 proposed transferring *D.peruensis* from the *guarani* group to a new one named *peruensis*. Vela and Rafael (2004) described three new species from the Pasochoa forest in Pichincha, Ecuador: *Drosophilaecuatoriana*, *D.pichinchana*, and *D.quitensis*. Later Vela and Rafael (2005) described *D.cuscungu*, which also became part of the *guarani* group. In a recent study Ratcov et al. (2017) described *D.butantan* and assigned *D.nigrifemur* Duda, 1927 to this group. An anthophilic species *D.peixotoi* was found breeding in the inflorescences of *Goeppertiamonophylla* by Vaz et al. (2018). This group is restricted to Central and South America (Kastritsis 1969).

Studies of the polytene chromosomes of some species of the *guarani* group (Kastritsis 1969) split of the *guarani* group into two subgroups, the *guarani* Dobzhansky & Pavan, 1943 subgroup with *D.ornatifrons*, *D.subbadia*, *D.guaru*, and the *guaramunu* King, 1947 subgroup with *D.maculifrons*, *D.griseolineata*, and *D.guaraja*. The *guarani* species group is probably not monophyletic which would invalidate this traditional subgroup division (Robe et al. 2002).

Most of these species are of medium size, have a brown coloration, but differ in shade and intensity. They have two prominent oral bristles of approximately equal length, pollinose mesonota and pleurae, and divergent anterior scutellar bristles. All species have strongly clouded cross veins in the wings except *D.guaraja* that has slightly clouded cross veins (King 1947a).

Our recent collections in southern Ecuador discovered five new species of *Drosophila* from the *guarani* group, which are described below: *Drosophilazamorana* sp. n., *Drosophilaquinarensis* sp. n., *Drosophilacaxarumi* sp. n., *Drosophilasachapuyu* sp. n. and *Drosophilamisi* sp. n. We discuss the similarities between these new species and the others in the *guarani* group.

## Materials and methods

The flies were collected in Loja and Zamora Chinchipe Provinces of Ecuador, in the cloud forests of the Podocarpus National Park and the surrounding areas. Collections were made at three localities, San Francisco Scientific Station at 2190m (3°59'16.7"S; 79°5'35"W) and Cajanuma 1 at 2675m (4°6'53.7"S; 79°10'54.6"W) and Cajanuma 2 at 2800m (4°6'58.9"S; 79°10'11.9"W). Fifteen fermented banana traps were placed in each location ten meters apart from each other and one meter above the base of the trees. Traps were made using recycled 500 ml plastic bottles and baited with banana pieces fermented with yeast 24 hours before placement.

Living flies were captured with an entomological aspirator and transferred to vials with gelatin-banana media (Rafael et al. 2000). Females were individually isolated to produce isofemale lines. Adult specimens were preserved in microcentrifuge tubes with ethanol (70–80%) and glycerin (100%) solution (Márquez Luna 2005). The fermented banana baits were put inside glass jars sealed with cotton plugs for transport to the laboratory where the baits were retained until the emergence of adults.

External morphology of each fly was examined under a stereomicroscope (Zeiss; Discovery V8). Male and female terminalia were dissected and placed in KOH (10%) and boiled for ten minutes; they were then placed in 60% glycerin for females and 100% glycerin for males. Terminalia were compared with literature descriptions to determine the new species. The new species were illustrated using a microscope (Zeiss-46 70 86) with a camera lucida (Zeiss-47 46 20 9900). Structure measurements were made using the software AXIO VISION V4. Descriptive terms and indices follow the system of Bächli et al. (2004).

The holotypes and paratypes of the new species have been deposited in the Museo de Zoología – Invertebrados, Pontificia Universidad Católica del Ecuador, Quito (**QCAZ-I**).

## Taxonomy

### Drosophila zamorana sp. n.

Taxon classificationAnimaliaDipteraLauxaniidae

http://zoobank.org/BFFCB2CF-365E-42D0-80CD-F4D98E26A595

**Figure 1.**
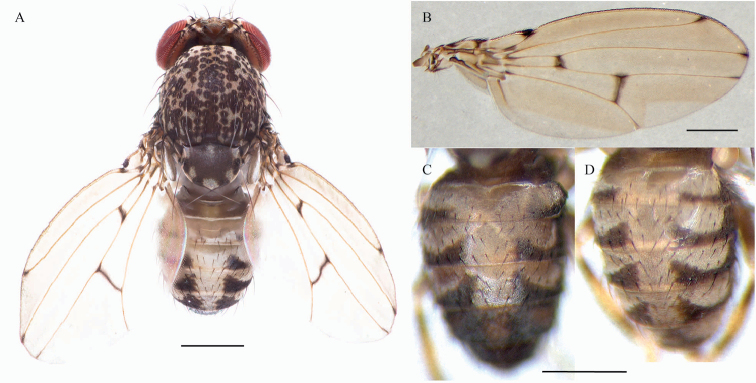
*Drosophilazamorana* sp. n., male holotype and female allotype external morphology. **A** Adult male, dorsal view **B** Male wing pigmentation **C, D** Abdomen, dorsal view, male and female respectively. Scale bars: 0.5 mm.

**Figure 2.**
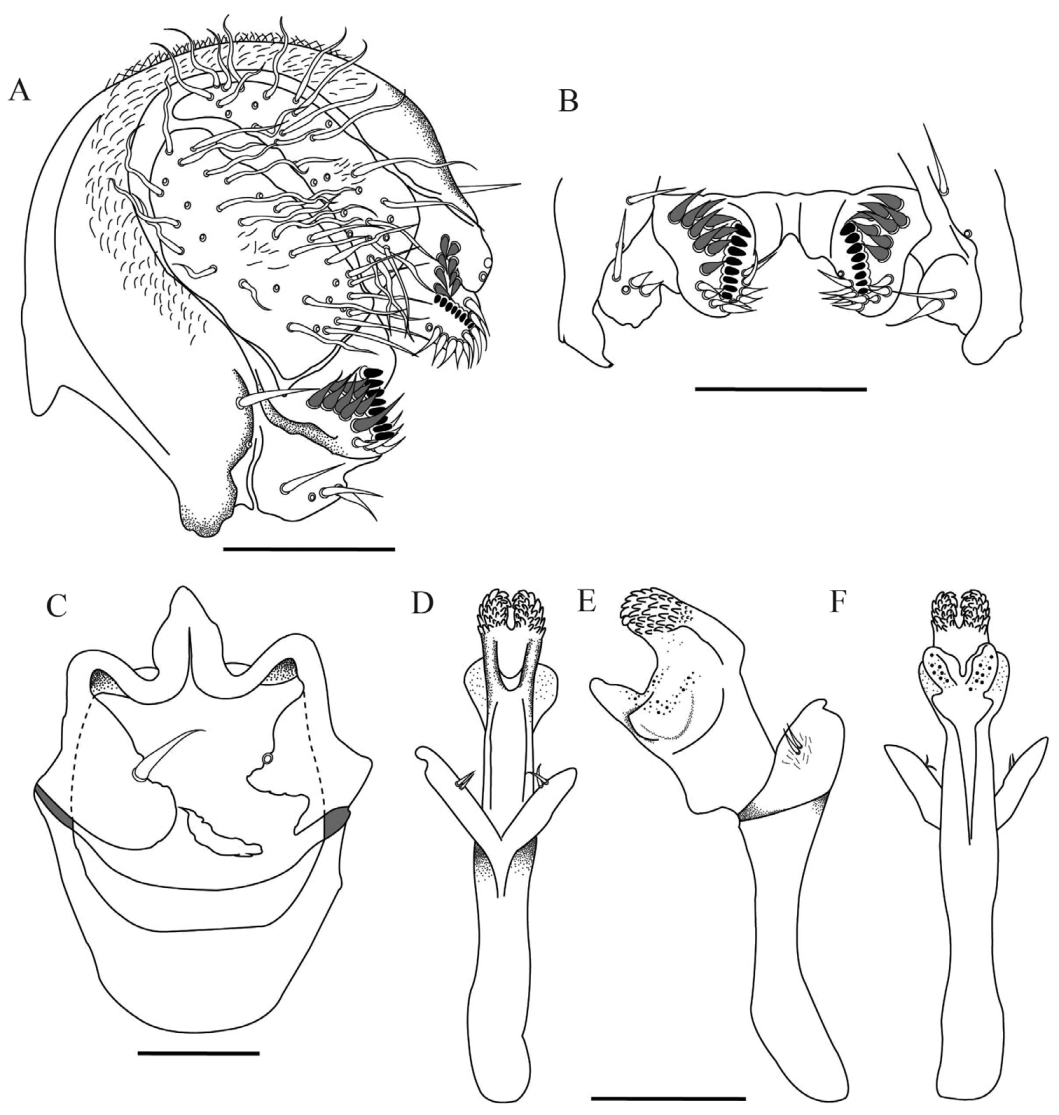
*Drosophilazamorana* sp. n., male terminalia of holotype. **A** Epandrium, cerci, surstylus **B** Decasternum **C** Hypandrium and gonopods in ventral view **D, E, F** Aedeagus and paraphyses in ventral, lateral and dorsal view, respectively. Scale bars: 100 µm.

**Figure 3.**
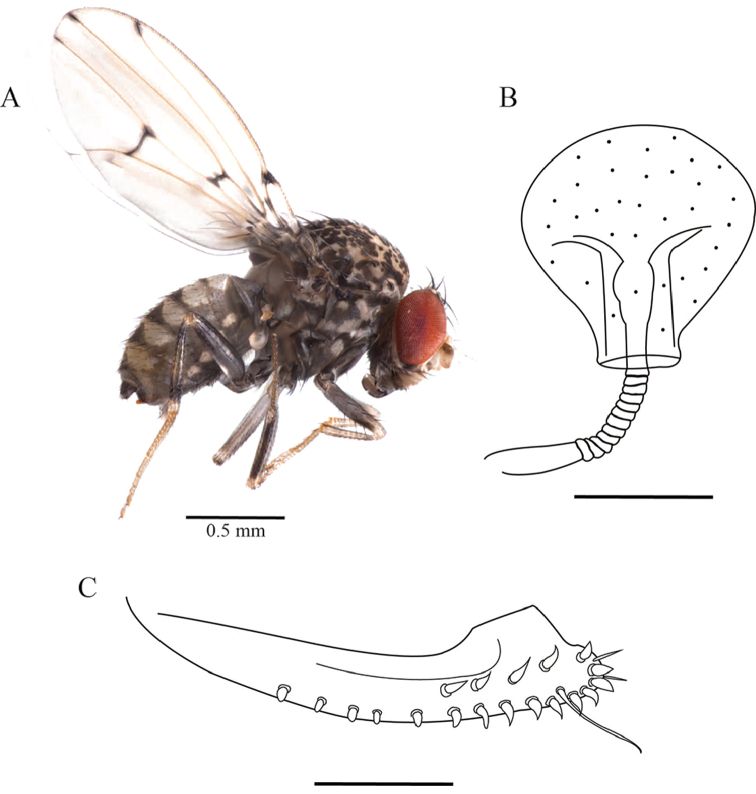
*Drosophilazamorana* sp. n., female external morphology and terminalia of allotype. **A** Adult female, lateral view **B** Spermatheca **C** Ovipositor. Terminalia scale bars: 100 µm.

Figs 1A–D , 2A–F , 3A–C

#### Type material.

**Holotype.** ♂ (dissected, terminalia in microvial), Ecuador, Zamora Chinchipe, San Francisco, 2190 m, 3°59'16.7"S; 79°5'35"W, IV.2015, Apr. 2015, A. Peñafiel col., A. Peñafiel & V. Rafael det. (QCAZ-I 3266). Allotype ♀ (dissected, terminalia in microvial), Ecuador, Zamora Chinchipe, San Francisco, 2190 m, 3°59'16.7"S; 79°5'35"W, Apr.2015, A. Peñafiel col., A. Peñafiel & V. Rafael det. (QCAZ-I 3267).

#### Paratypes.

9 ♂♂ and 9 ♀♀ (dissected, terminalia in microvial, descendants of isofemale line), Ecuador, Zamora Chinchipe, San Francisco, 2190 m, same data as holotype, A. Peñafiel col., A. Peñafiel & V. Rafael det. (QCAZ-I 3268-3285).

#### Diagnosis.

Aristae generally with five dorsal and three ventral branches, plus terminal fork. Two prominent equally long oral bristles. Thorax brown, with bristles arising from dark brown spots, scutellum brown with some irregular light spots. Wings beige, veins bM-Cu and dM-Cu strongly clouded tips of R2 + 3, R4 + 5 radial and M veins darkened. Abdomen dark brown, 1^st^ tergite beige, 2^nd^–4^th^ tergites of males with dorsal midline and white butterfly-shaped areas, followed by a white rounded area at the edge of each tergite; 5^th^ and 6^th^ tergites dark brown. Cerci not fused to epandrium. Hypandrium shield-shaped. Gonopod elongated, bearing one long bristle. Aedeagus voluminous with two ventral sheets covered in spines, dorsally with two membranous sheets covered with tiny spines studs. Paraphyses triangular, bearing two small bristles.

#### Description.

***Male.*** Holotype external morphology: total length (body + wings) 3.08 mm, body length 2.50 mm. Body color dark brown (Figure 1A).

*Head*. Aristae with five dorsal and three ventral branches, plus terminal fork. Frons dark brown, frontal length 0.20 mm; frontal index = 0.73; top to bottom width ratio = 1.44. Medial vertical seta was closer to lateral vertical seta and slightly towards the outer edge of the orbital plate; distance of or3 to or1 = 0.08, distance of or3 to vtm = 0.09; or1-or3 ratio = 1.7, or2-or1 ratio = 0.23, distance of ocellar setae = 1 of frontal length, distance of postocellar setae = 0.64 of frontal length. vt index = 0.66. Ocellar triangle dark brown, 48% of frontal length, ocellus yellow; frontal triangle brown. Frontal vitta yellowish brown. Gena and postgena dark brown. Carina not sulcate. Cheek index = 7.5. Eyes wine red; eye index = 1.4. Two prominent equally long oral bristles; vibrissa index = 1.1.

*Thorax*. Brown, with bristles arising from dark brown spots, thorax length 0.80 mm, acrostichal hairs in six rows between the two anterior dorsocentral setae, h index = 0.81. Transverse distance of dorsocentral setae = 2.28, dc index = 0.87. Scutellum brown with some irregular white spots (Figure 1A), distance between apical scutellar seta = 1, scut index = 1.13; medial katepisternal seta one third of the previous, sterno index = 2.16. Legs light brown, coxae and femora dark brown, tibiae with two brown rings. *Wings* beige, alar length 2.10 mm, width 0.90 mm, veins bM-Cu and dM-Cu strongly clouded tips of R2+3, R4+5 radial and M veins darkened. First costal section apex black (Figure 1B). Indices: alar = 2.25; C = 3.61; ac = 2.29; hb = 0.53; 4c = 0.86; 4v = 2.08; 5x = 1.65; M = 0.73 and prox. x = 0.77.

*Abdomen*. Dark brown, 1^st^ tergite beige, 2^nd^ – 5^th^ tergites of males with dorsal midline and white butterfly-shaped areas with posterior dark pigmentation which reaches the exterior margin, leaving a white rounded laterally area; 6^th^ tergite dark brown (Figure 1C).

*Male terminalia*. Epandrium dorsally microtrichose with two lower and no upper bristles, four bristles on the ventral lobe. Cerci sclerotized and not fused to epandrium, with some microtrichose areas in the middle (Figure 2A). Surstylus triangular with a row of eight primary teeth, nine secondary pointed teeth on the right and eight on the left; nine marginal bristles on the right and ten on the left (Figure 2B). Hypandrium sclerotized in shield-shape. Gonopod elongated bearing one long bristle (Figure 2C).

*Aedeagus*. Sclerotized, voluminous with two ventral sheets covered in short spines, dorsally with two membranous sheets covered with tiny spines. Paraphyses triangular and slightly microtrichose, bearing two small bristles (Figure 2D-F).

Variation in paratypes (dry mounted specimens). Total length (body + wings) 2.95–3.12 mm, body length 2.35–2.6 mm. Head. Frontal length 0.24–0.34 mm, frontal index = 0.73–1.12, top to bottom width ratio = 1.5–2.04; distance of or3 to or1 = 0.07–0.1, distance of or3 to vtm = 0.9–0.11, or1-or3 ratio = 0.52–0.94, or2-or1 ratio = 0.18–0.42, distance of ocellar setae = 0.5–1.04 of frontal length, distance of postocellar setae = 0.46–0.7 of frontal length, vt index = 0.96–1.21; cheek index = 7.0–9.8; vibrissa index = 0.92–1.12; eye index = 1.15–1.68. Thorax. Length 0.64–0.77 mm, h index = 0.85–1.04. Transverse distance of dorsocentral setae = 1.68–2.46, dc index = 0.69–0.70. Distance between apical scutellar seta = 0.94–1.05. scut index = 0.87–1.13; sterno index = 0.94–2.87.

***Female.*** Allotype and paratypes (isofemale descendants). Allotype: total length (body + wings) 3.24 mm, body length 2.43 mm. Body color dark brown (Figure 3A).

*External morphology*. Same as the male except abdomen. Abdomen 1^st^ tergite beige, 2^nd^ – 6^th^ tergites with dorsal midline and white butterfly-shaped areas with posterior dark pigmentation which reaches the exterior margin, leaving a white rounded lateral area (Figure 1D). Wings beige, alar length 2.54 mm, width 1.07 mm, veins bM-Cu and dM-Cu strongly clouded tips of R2+3, R4+5 radial and M veins darkened. First costal section apex black. Indices: alar = 2.37; C = 4.04; ac = 2; hb = 0.42; 4c = 0.73; 4v = 1.98; 5x = 1.88; M = 0.56 and prox. x = 0.82.

*Terminalia*. Spermatheca lightly pigmented, bulb-shaped, covered with tiny spines, duct partially invaginated (Figure 3B). Ovipositor elongated, sclerotized, with 13 marginal and six discal teeth, one long bristle, and two fine hairs (Figure 3C).

Variation in paratypes (dry mounted specimen). Head. Frontal length 0.23–0.27 mm, frontal index = 0.6–0.71, top to bottom width ratio = 1.31–1.36; distance of or3 to or1 = 0.07–0.09, distance of or3 to vtm = 0.09–0.12, or1-or3 ratio = 0.8–1.0, or2-or1 ratio = 0.22–0.57, distance of ocellar setae = 1.0–1.3 of frontal length, distance of postocellar setae = 0.60–0.68 of frontal length, vt index = 0.77–1.0; cheek index = 5.62–7.83; vibrissa index = 0.85–0.94; eye index = 1.20–1.27. Thorax length 0.81–0.89 mm, h index = 0.86–1.0. Transverse distance of dorsocentral setae = 2.17–3.0, dc index = 0.70–0.83. Distance between apical scutellar seta = 1. scut index = 1.08–1.25; sterno index = 0.92–0.95.

#### Etymology.

Named in recognition of the collection site, Zamora Chinchipe province of Ecuador.

#### Distribution.

Known only from the type locality.

#### Ecology.

Unknown. The type specimen was found in the banana-bait traps placed at the locality, which suggests that this species feeds on fermented fruits as do many other *Drosophila* species. This species has been reared with gelatin banana media devised by Rafael et al. (2000). The habitat is a relatively well-preserved montane forest.

#### Relationship to other species.

This species belongs to the *guarani* species-group. The most similar species are *Drosophilaurubamba* Vilela & Pereira, 1993 and *Drosophilatucumana* Vilela & Pereira, 1985. *Drosophilazamorana* sp. n. is similar to *D.urubamba* but the most important difference is in the aedeagus. First, the distal end of the aedeagus in *D.zamorana* is narrower than in *D.urubamba*. Second, the form of the aedeagus in *D.urubamba* is bulkier than in *D.zamorana*. There is a little difference in the paraphyses; *D.zamorana* has some microtrichose areas surrounding the two small bristles that *D.urubamba* does not have. These differences distinguish these two species, mainly because of the cryptic external morphology.

### Drosophila quinarensis sp. n.

Taxon classificationAnimaliaDipteraLauxaniidae

http://zoobank.org/CAA65626-0E02-4464-A97B-187887EE1F08

Figs 4A–F , 5A–B

#### Type material.

Holotype ♂ (dissected, terminalia in microvial), Ecuador, Loja, Cajanuma, 2800 m, 4°6'58.9"S; 79°10'11.9"W, 19 Nov. 2015, A. Peñafiel col., A. Peñafiel & V. Rafael det. (QCAZ-I 3286). Allotype ♀ (dissected, terminalia in microvial), Ecuador, Loja, Cajanuma, 2800 m, 4°6'58.9"S; 79°10'11.9"W, 19 Nov. 2015, A. Peñafiel col., A. Peñafiel & V. Rafael det. (QCAZ-I 3287).

#### Paratypes.

6 ♂♂ and 1 ♀♀ (dissected, terminalia in microvial, descendants of isofemale line), Ecuador, Loja, Cajanuma, 2800 m, 4°6'58.9"S; 79°10'11.9"W, 19 Nov. 2015, A. Peñafiel col., A. Peñafiel & V. Rafael det. (QCAZ-I 3288–3293, 3297). 3♀ (dissected, terminalia in microvial, descendants of isofemale line), Ecuador, Loja, Cajanuma, 2675 m, 4°6'53.7"S; 79°10'54.6"W, 19 Nov. 2015, A. Peñafiel col., A. Peñafiel & V. Rafael det. (QCAZ-I 3298–3300).

#### Diagnosis.

Aristae with four dorsal and two ventral branches. Two prominent oral bristles, the second slightly shorter than the first. Thorax brown. Abdomen yellow, 2^nd^ – 5^th^ tergites with dorsal midline, with posterior dark pigmentation which reaches and covers the exterior margin. Cerci not fused to epandrium. Aedeagus voluminous, with two ventral asymmetrical projections covered in short spines and two dorsal projections with tiny spines. Hypandrium is shield-shaped with a sclerotized edge. Gonopod bearing one bristle. Paraphyses strongly sclerotized and joined to gonopod, bearing two small bristles.

#### Description.

***Male.*** Holotype external morphology: total length (body + wings) 3.38 mm, body length 2.36 mm. Body color dark brown.

*Head*. Aristae with four dorsal and two ventral branches, plus terminal fork and fine hairs. Orbital plate yellowish brown, frontal length 0.29 mm; frontal index = 0.96; top to bottom width ratio = 1.7. Medial vertical seta was closer to lateral vertical seta and slightly towards the outer edge of the orbital plate, distance of or3 to or1 = 0.11, distance of or3 to vtm = 0.12, or1-or3 ratio = 1.30, or2-or1 ratio = 0.35, distance of postocellar setae = 0.68 of frontal length. vt index = 0.85. Ocellar triangle brown, 48.27% of frontal length, ocellus yellow, frontal vitta yellowish brown. Two prominent oral bristles, second slightly shorter than the first. Carina not sulcate.

*Thorax*. Brown, acrostichal hairs in six rows between the two anterior dorsocentral setae that decrease in number towards the rear. Medial katepisternal seta smaller than the previous seta. *Wings* yellow. Alar length 2.62 mm, alar width 1.29 mm. Vein dM-Cu clouded. Alar indices: alar = 1.07; C = 5.62; ac = 1.37; 4c = 0.43; 4v = 1.25; 5x = 1.20; M = 0.40 and prox. x = 0.37. Legs yellow, femora and tibiae dark brown, metatarsi and tarsi light brown.

**Figure 4.**
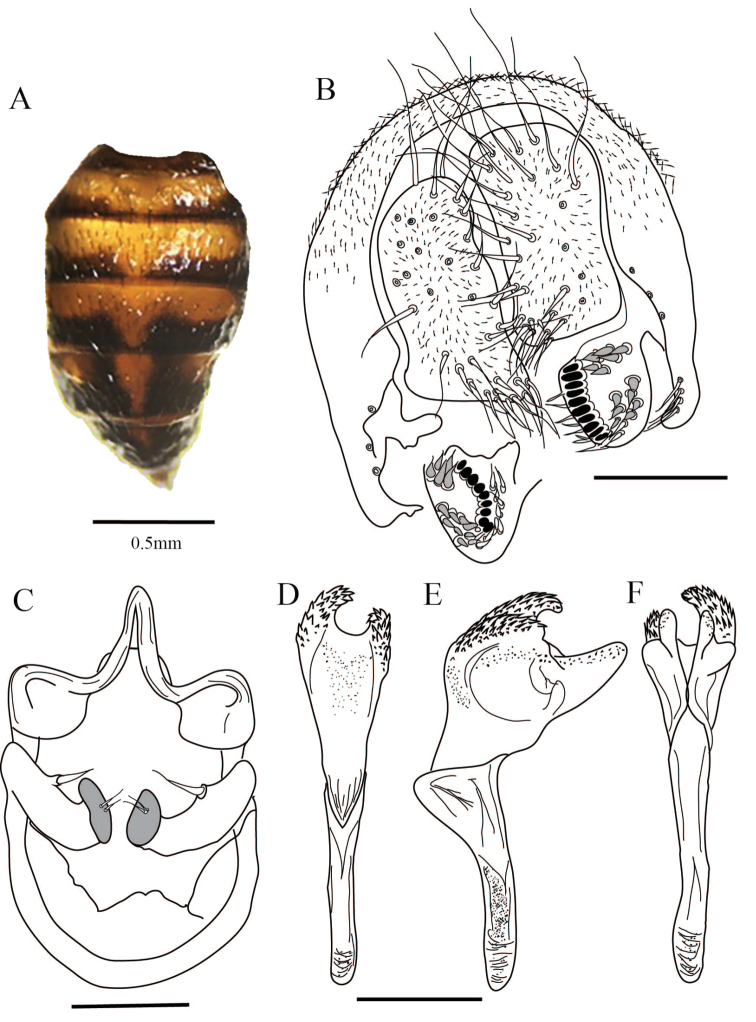
*Drosophilaquinarensis* sp. n., abdomen and male terminalia of holotype. **A** Male abdomen, dorsal view **B** Epandrium, cerci, surstylus and decasternum **C** Hypandrium, gonopods and paraphyses, ventral view **D, E, F** Aedeagus in ventral, lateral and dorsal view, respectively. Terminalia scale bars: 100 µm.

*Abdomen*. Yellow, 1^st^ tergite brown, 2^nd^ – 5^th^ tergites with dorsal midline and posterior dark pigmentation which reaches and covers the exterior margin, 6^th^ tergite dark (Figure 4A).

*Male terminalia*. Epandrium strongly sclerotized, microtrichose, with seven lower and no upper bristles, four bristles on the ventral lobe. Cerci microtrichose not fused to epandrium. Surstylus oval with nine primary teeth on the right and ten on the left; sharp secondary teeth strongly sclerotized, in two groups, five upper and nine lower on the right, six upper and seven lower on the left; with eleven marginal bristles (Figure 4B). Hypandrium in shield-shape, with edge strongly sclerotized. Gonopod elongated bearing one bristle (Figure 4C).

*Aedeagus*. Aedeagus voluminous, with two ventral asymmetrical projections covered in short spines and two dorsal projections with bright tiny spines. Primitive ventral rod. Aedeagal apodeme slightly sclerotized (Figure 4D–F). Paraphyses joined to gonopod strongly sclerotized bearing two small bristles (Figure 4C).

Variation in paratypes (dry mounted specimens). Total length (body + wings) 4.19–4.73 mm, body length 1.96–3.01 mm. Head. Frontal length 0.27–0.42 mm, frontal index = 0.66–0.85, top to bottom width ratio = 1.20–1.38; distance of or3 to or1 = 0.11–0.14, distance of or3 to vtm = 0.12–0.13, or1-or3 ratio = 0.85–1.05, or2-or1 ratio = 0.19–0.34, distance of ocellar setae = 0.69–1.03 of frontal length, distance of postocellar setae = 0.84–0.85 of frontal length, vt index = 0.87–1.12; cheek index = 6.77–8.42; vibrissa index = 0.86–1.05; eye index = 1.17–1.51. Thorax length 0.89–1.07 mm, h index = 0.84–1.21. Transverse distance of dorsocentral setae = 1.91–2.55, dc index = 0.84–0.87. Distance between apical scutellar seta = 0.95–1.09. scut index = 1.11–1.39; sterno index = 1–1.04.

***Female.*** Allotype and paratypes (isofemale descendants). Allotype: total length (body + wings) 5.11 mm, body length 3.08 mm. Body color dark brown.

External morphology. Same as the male.

**Figure 5.**
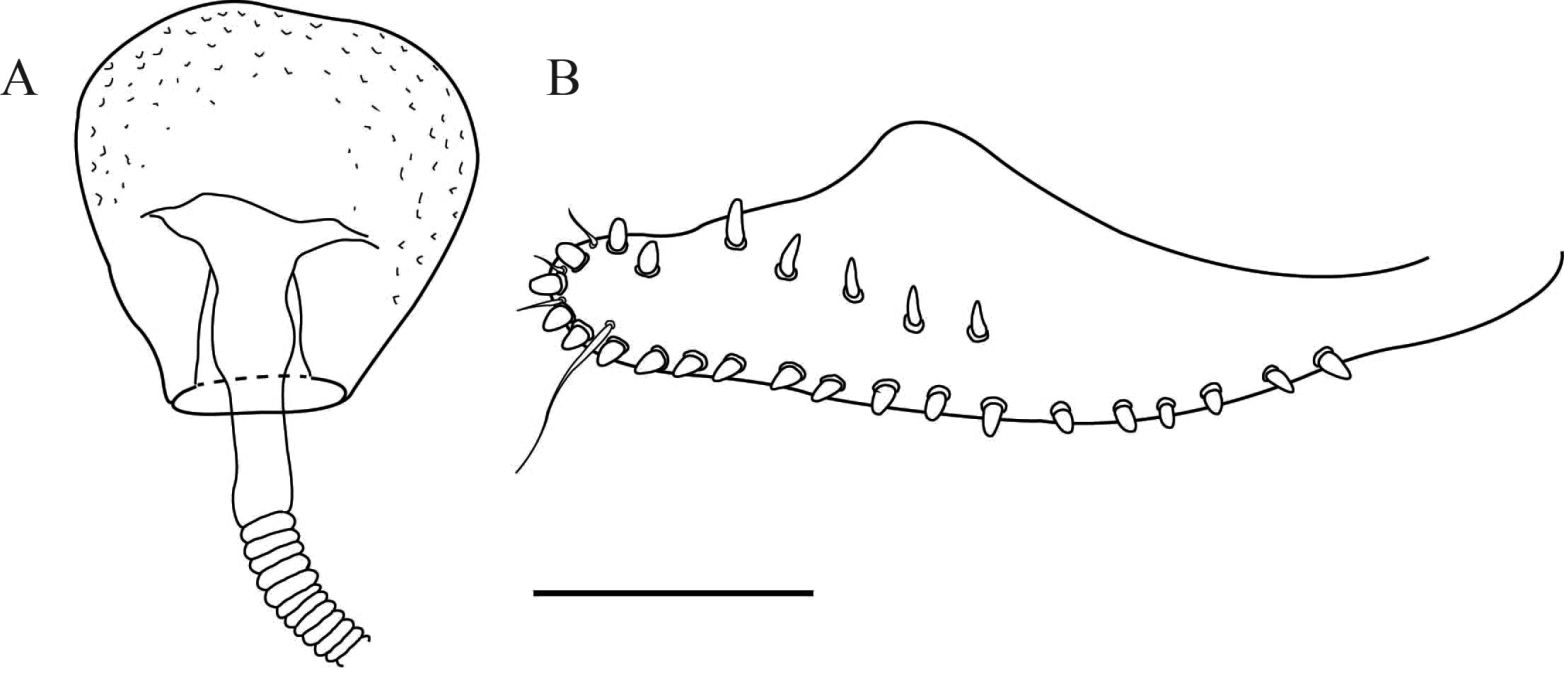
*Drosophilaquinarensis* sp. n., female terminalia of allotype. **A** Spermatheca **B** Ovipositor. Scale bar: 100 µm.

*Wings* yellow. Alar length 3.7 mm, alar width 1.65 mm. Vein dM-Cu clouded. Alar indices: alar = 2.24; C = 4.27; ac = 1.87; hb = 0.32; 4c = 0.56; 4v = 1.54; 5x = 1.22; M = 0.42 and prox. x = 0.39.

Like male except yellow abdomen, 1^st^ tergite brown, 2^nd^ – 6^th^ tergites with dorsal midline and white butterfly-shaped areas with posterior dark pigmentation which reaches the exterior margin, leaving a white rounded lateral area.

*Terminalia*. Spermatheca lightly pigmented, bulb-shaped, with small indents, duct partway invaginated (Figure 4A). Ovipositor elongated, sclerotized, with 19 marginal and seven discal teeth, one long bristle, and three fine hairs (Figure 4B).

Variation in paratypes (dry mounted specimens). Total length (body + wings) 3.13–5.11 mm, body length 2.3–3.08 mm. Head. Frontal length 0.4 mm, frontal index = 0.81–0.85, top to bottom width ratio = 1.18–1.40; distance of or3 to or1 = 0.13–0.15, distance of or3 to vtm = 0.07–0.15, or1-or3 ratio = 0.54–0.62, or2-or1 ratio = 0.35–0.54, distance of ocellar setae = 0.85–0.87 of frontal length, distance of postocellar setae = 0.72–0.73 of frontal length, vt index = 0.85–1.08; cheek index = 7.35–10.16; vibrissa index = 0.84–1.11; eye index = 1–1.27. Thorax. Length 1.05–1.08 mm, h index = 0.94–1.06. Transverse distance of dorsocentral setae = 1.77–2.18, dc index = 0.68–0.70. Distance between apical scutellar setae = 0.77–0.96. scut index = 0.96–1.33; sterno index = 0.92–1.24.

#### Etymology.

Name refers to the Quinara district in Loja Province of Ecuador. In this district there is a legend that the treasure demanded by the Spaniards to release Atahualpa from captivity is still hidden among the old farms in the region.

#### Distribution.

Known only from the type locality.

#### Ecology unknown.

The type specimen was found in the banana-bait traps placed at the locality, which suggests that this species feeds on or breeds in fermented fruits as do many other *Drosophila* species. The habitat is a well-preserved montane forest.

#### Relationship to other species.

The general shape of the male terminalia, especially the asymmetrical projections of the aedeagus, suggests a close relationship to *Drosophilaquitensis* Vela & Rafael, 2004. The main difference is in the aedeagus. The edeagus of *D.quinarensis* is more elongate than in *D.quitensis*.

### Drosophila caxarumi sp. n.

Taxon classificationAnimaliaDipteraLauxaniidae

http://zoobank.org/F1912DEF-831A-4BB0-86EA-992F73AE57C8

**Figure 6.**
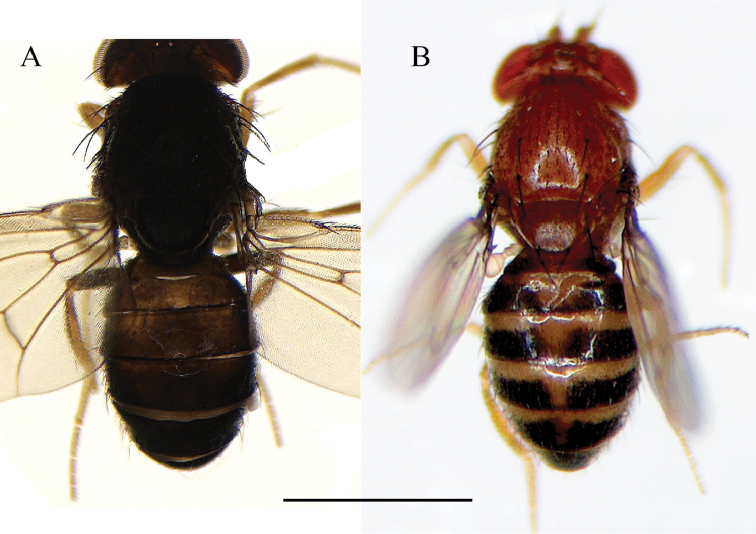
**A***Drosophilacaxarumi* sp. n., male holotype external morphology, dorsal view. **B***Drosophilaguaraja* King, from Zamora Chinchipe, Ecuador, male external morphology, dorsal view. Scale bar: 1 mm.

**Figure 7.**
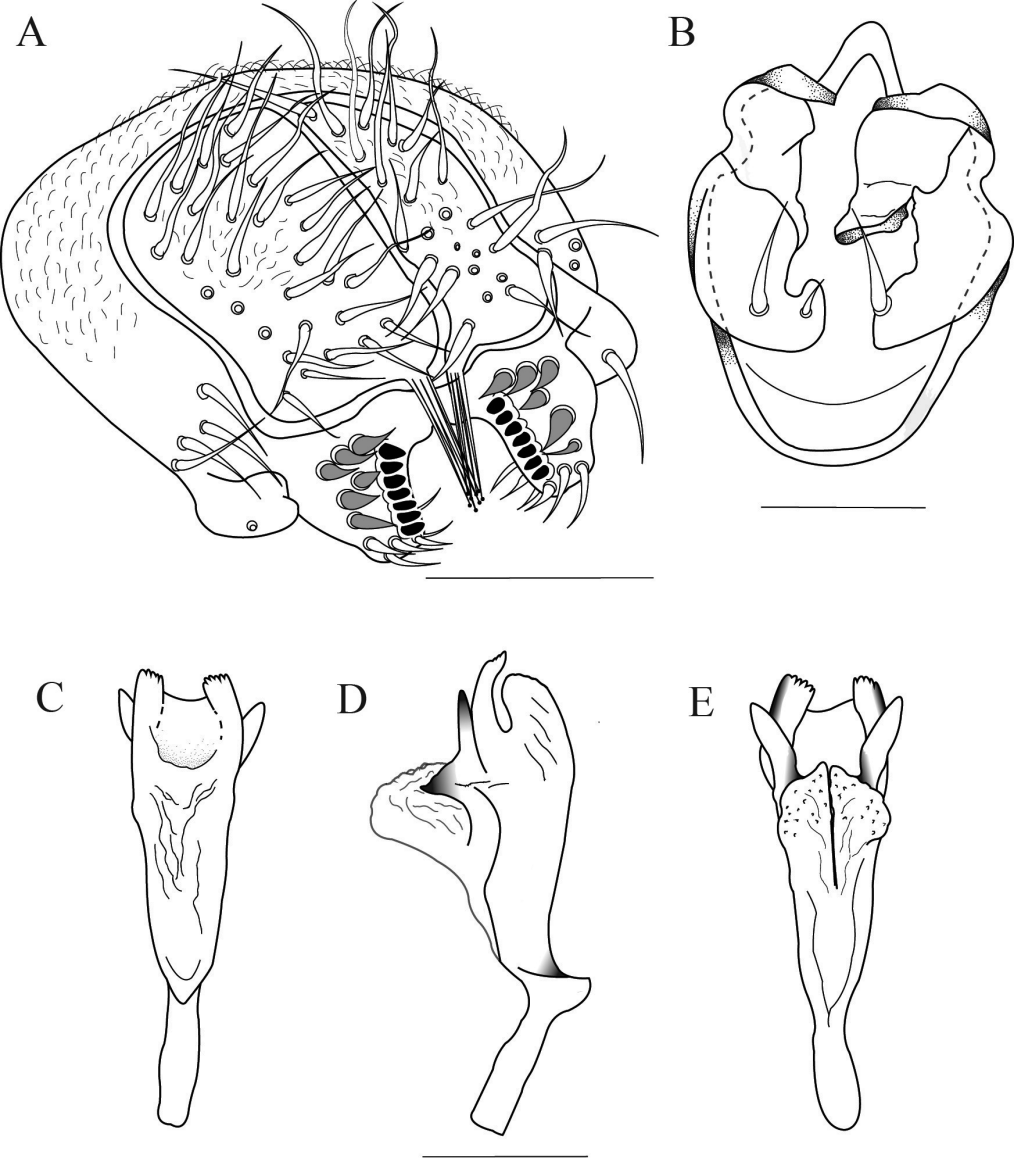
*Drosophilacaxarumi* sp. n., male terminalia of holotype. **A** Epandrium, cerci, surstylus, and decasternum **B** Hypandrium, gonopods and paraphyses, ventral view **C, D, E** Aedeagus in ventral, lateral and dorsal view, respectively. Scale bars: 100 µm.

**Figure 8.**
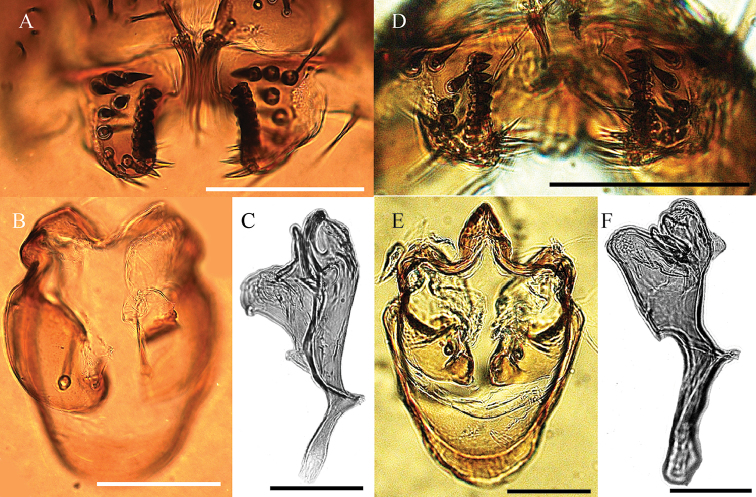
*Drosophilacaxarumi* sp. n., male terminalia of holotype. **A** Surstylus, and decasternum **B** Hypandrium, gonopods and paraphyses, ventral view **C** Aedeagus in lateral view. *Drosophilaguaraja* King, from Zamora Chinchipe, Ecuador. **D** Surstylus, and decasternum **E** Hypandrium, gonopods and paraphyses, ventral view **F** Aedeagus in lateral view. Scale bars: 100 µm.

Figs 6A–B , 7A–E , 8A–F

#### Type material.

Holotype ♂ (dissected, terminalia in microvial), Ecuador, Loja, Cajanuma, 2675 m, 4°6'53.7"S; 79°10'54.6"W, 19 Nov. 2015, A. Peñafiel col., A. Peñafiel & V. Rafael det. (QCAZ-I 3306).

#### Paratypes.

2 ♂♂ (dissected, terminalia in microvial), Ecuador, Napo, Río Guango, 2548 m, 00°32'14.0"S; 77°57'13.4"W, 19 Sep. 2015, A. B. Manzano col., A. Peñafiel & V. Rafael det. (QCAZ-I 3307–3308).

#### Diagnosis.

Body color yellow. Aristae with four dorsal and two ventral branches. Two prominent oral bristles. Thorax brown. Clear wings. Aedeagus with two dorsal membranes covered in microprojections which continue to a sclerotized triangular projection and another distally serrated sheet joined ventrally by a membrane. Hypandrium in shield-shape. Gonopod fused to paraphyses, bearing a long bristle and short bristle.

#### Description.

***Male.*** Holotype external morphology: total length (body + wings) 2.90 mm, body length 2.30 mm. Body color brown.

*Head*. Aristae with four dorsal and two ventral branches plus terminal fork and small hairs. Orbital plate yellowish brown; frontal length 0.25 mm, frontal index = 1.0; top to bottom width ratio = 1.68. Medial vertical seta closer to lateral vertical seta and slightly towards the outer edge of the orbital plate. Distance of or3 to or1 = 0.06, distance of or3 to vtm = 0.09, or1-or3 ratio = 0.59; or2-or1 ratio = 0.30. Ocelar triangle yellow, distance of postocellar setae = 0.52 of frontal length, ocellus yellow; frontal vitta yellow. Two prominent oral bristles, vibrissa index = 0.85. Cheek index = 3.33. Carina not sulcate. Eyes wine red, eye index = 1.30.

*Thorax*. Brown (Figure 6A), thorax length 0.66 mm. h index = 1.18. Transverse distance of dorsocentral setae = 1.53, dc index = 0.81. Distance between apical scutellar seta = 1.38, scut index = 0.93, sterno index could not be calculated (broken bristles in holotype). Clear wings. Alar length 1.65 mm, alar width 0.86 mm. Alar indices: alar index = 1.91; C = 3.37; ac = 1.94; hb = 0.25; 4c = 0.89; 4v = 2.25; 5x = 1.44; M = 0.66 and prox. x = 0.46.

*Abdomen*. Yellow, 1^st^ – 6^th^ tergites with dark brown pigmentation that covers entirely each tergite (Figure 6A).

*Male terminalia.* Epandrium microtrichose with four lower and no upper bristles, one bristle on the ventral lobe. Cerci not fused to epandrium, microtrichose, tip bearing four cotton swab-shaped bristles. Surstylus rectangular with seven primary teeth, six secondary sharp teeth on the right and five on the left, six marginal bristles (Figs 7A, 8A). Hypandrium in shield-shape and sclerotized. Gonopod elongated, fused to the paraphyses, bearing a long bristle and other short one (Figs 7B, 8B).

*Aedeagus*. Sclerotized, with two dorsal membranes covered in microprojections which continue to a sclerotized triangular projection and another distally serrated sheet joined ventrally by a slightly sclerotized membrane (Figs 7C–E, 8C).

Variation in paratypes (dry mounted specimen). Cannot be calculated, broken bristles in paratypes.

#### Etymology.

Name refers to the *Caxarumi* district in Loja Province of Ecuador. In Kichwa, *caxa* refers to *Chakka* (possum) and *rumi* (stone, rock).

#### Distribution.

*Drosophilacaxarumi* is known from two localities (elevation range is 2548–2675 m) from Loja Province, Podocarpus National Park and Napo Province, Río Guango.

#### Ecology.

Unknown. The type specimen was found in the fermented banana-bait traps placed at the locality, which suggests that this species feeds or breeds on fermented fruit as many other *Drosophila* species. The habitat is a well-preserved intermediate montane forest.

#### Relationship to other species.

The most similar species to *Drosophilacaxarumi* sp. n. is *D.guaraja* King, 1947b. We compared *D.caxarumi* with descendants of *D.guaraja* reared from an isofemale line named ZBCI036 from Podocarpus National Park, Bombuscaro, 1000 msnm. These two species share the same cotton swab-shaped bristles on the ventral side of the cerci (Figure 8A, D). *Drosophilacaxarumi* and *D.guaraja* differ in the general shape of the aedeagus (Figure 8C, F). In *D.caxarumi* the aedeagus is bent towards the dorsal side. In *D.guaraja* the paraphyses are microtrichose while in *D.caxarumi* they are smooth and hairless. Both species differ markedly in external morphology (Figure 6A, B), body color of *D.caxarumi* is mainly brown while in *D.guaraja* is yellow. *D.guaraja* abdomen is yellow with broad, dark brown bands different from the brown abdomen in *D.caxarumi* (Figure 6A, B).

### Drosophila sachapuyu sp. n.

Taxon classificationAnimaliaDipteraLauxaniidae

http://zoobank.org/C4F4A33E-ED73-44E3-A87A-6BE026A2DD93

Figs 9A–F , 10A–C

#### Type material.

Holotype ♂ (dissected, terminalia in microvial), Ecuador, Loja, Cajanuma, 2675 m, 4°6'53.7"S; 79°10'54.6"W, 23 Apr. 2015, A. Peñafiel col., A. Peñafiel & V. Rafael det. (QCAZ-I 3309). Allotype ♀ (dissected, terminalia in microvial), Ecuador, Loja, Cajanuma, 2675 m, 4°6'53.7"S; 79°10'54.6"W, 23 Apr. 2015, A. Peñafiel col., A. Peñafiel & V. Rafael det. (QCAZ-I 3310).

#### Paratypes.

9 ♂♂ and 9 ♀♀ (dissected, terminalia in microvial, descendants of isofemale line), Ecuador, Loja, Cajanuma, 2675 m, 4°6'53.7"S; 79°10'54.6"W, 23 Apr. 2015, A. Peñafiel col., A. Peñafiel & V. Rafael det. (QCAZ-I 3311–3328).

#### Diagnosis.

Body color yellow. Aristae with six dorsal and two ventral branches. Two prominent oral bristles. Thorax yellow. Wings clear. Cerci not fused to epandrium. Aedeagus with two sheets fused by a membrane with tiny spines, with a ventral blade with wavy edge. Hypandrium in shield-shape. Gonopod bearing one long bristle. Paraphyses fused to gonopod bearing two small bristles.

#### Description.

***Male.*** Young specimen. Holotype external morphology: total length (body + wings) 3 mm, body length 2.40 mm. Body color yellow.

*Head*. Aristae with six dorsal and two ventral branches plus terminal fork and fine hairs. Orbital plate yellowish brown, frontal length 0.29 mm; frontal index = 0.82; top to bottom ratio = 0.14; Medial vertical seta closer to lateral vertical seta. Distance of or3 to or1 = 0.11, distance of or3 to vtm = 0.09, or1-or3 ratio = 0.62; or2-or1 ratio = 0.47, vt index = 0.84. Ocellar triangle yellowish brown, ocellus yellow, distance of ocellar setae = 0.51 of frontal length, distance of postocellar setae = 0.75 of frontal length; frontal vitta yellow. Two prominent oral bristles of the same size, vibrissa index = 0.84. Cheek index = 4.58. Carina not sulcate. Eyes wine red, eye index = 1.28.

*Thorax*. Yellow, thorax length 0.60 mm, acrostichal hairs in eight rows between the two anterior dorsocentral setae that decrease in number toward the rear. h index = 1.2. Transverse distance of dorsocentral setae = 2.17, dc index = 0.65. Distance between apical scutellar seta = 1.16, scutellum yellow, scut index = 1.34. Medial katepisternal seta three quarters of the length of the anterior seta, sterno index = 2.2. Clear wings. Alar length 2.29 mm, alar width 1.09 mm. Alar indices: alar = 2.10; C = 2.76; ac = 2.04; hb = 0.30; 4c = 0.47; 4v = 1.25; 5x = 1.5; M = 0.4 and prox. x = 0.31.

**Figure 9.**
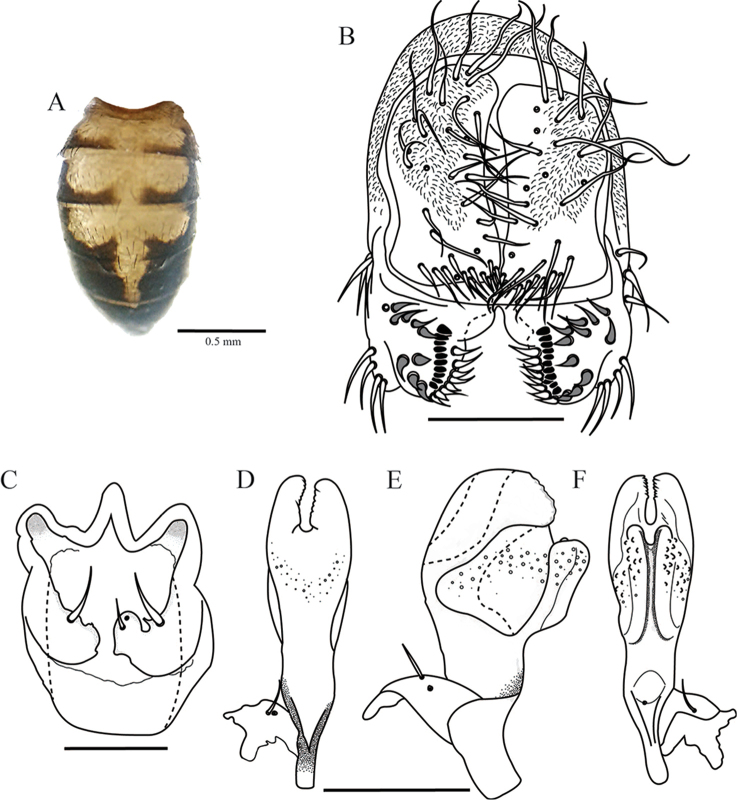
*Drosophilasachapuyu* sp. n., abdomen and male terminalia of holotype. **A** Male abdomen, dorsal view **B** Epandrium, cerci, surstylus and decasternum **C** Hypandrium, gonopods and paraphyses, ventral view **D, E, F** Aedeagus and paraphyses in ventral, lateral and dorsal view, respectively. Terminalia scale bars: 100 µm.

*Abdomen*. Yellow with dorsal midline that reaches the 5^th^ tergite, 1^st^ tergite yellowish brown, 2^nd^ – 4^th^ tergites with posterior dark pigmentation which reaches and covers the exterior margin leaving a round clear area, 5^th^ tergite with dark pigmentation which covers the entire tergite, 6^th^ tergite totally dark (Figure 9A).

*Male terminalia.* Epandrium microtrichose with three lower and no upper bristles, four bristles on the ventral lobe. Cerci not fused to epandrium. Surstylus triangular, with ten primary teeth on the right and eleven on the left side, ten sharp secondary teeth on the right and eleven on the left side, eight marginal bristles on each side (Figure 9B). Hypandrium in shield-shape and membranous. Gonopod elongated bearing one long bristle (Figure 9C).

*Aedeagus*. Young specimen slightly sclerotized, ends in two sheets fused by a membrane with small bright tiny spines, with a wider blade with wavy edge. Aedeagus apodeme broken. Paraphyses fused to gonopod bearing two small bristles (Figure 9 D-F).

Variation in paratypes (dry mounted specimen). Total length (body + wings) 3.82–4.43 mm, body length 2.49–3.08 mm. Head. Frontal length 0.28–0.32 mm, frontal index = 0.7–0.85, top to bottom width ratio = 1.08–1.59; distance of or3 to or1 = 0.07–0.12, distance of or3 to vtm = 0.11–0.14, or1-or3 ratio = 0.86–1.40, or2-or1 ratio = 0.20–0.38, distance of ocellar setae = 0.83–1.17 of frontal length, distance of postocellar setae = 0.68–0.96 of frontal length, vt index = 0.77–0.93; cheek index = 5.36–10.66; vibrissa index = 0.86–1.07; eye index = 1.08–1.40. Thorax length 0.76–0.98 mm, h index = 0.69–1.27. Transverse distance of dorsocentral setae = 1.0–2.47, dc index = 0.61–1.06. Distance between apical scutellar seta = 0.76–1.0, scut index = 1.10–1.51; sterno index = 1.28–2.60.

***Female.*** Allotype and paratypes (isofemale descendants). Allotype: total length (body + wings) 4.41 mm, body length 2.94 mm. Body color dark brown.

**Figure 10.**
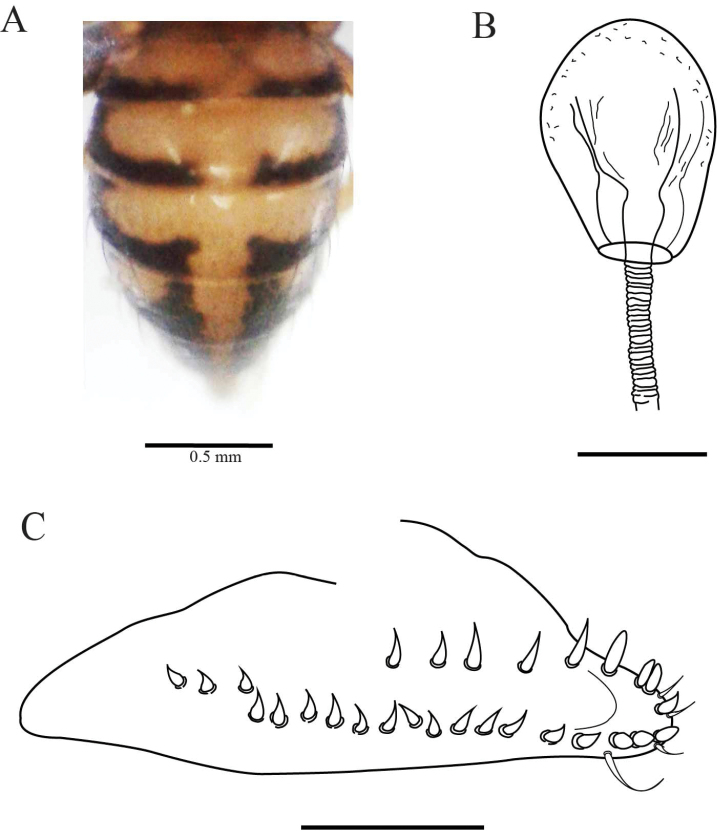
*Drosophilasachapuyu* sp. n., abdomen and female terminalia of allotype. **A** Female abdomen, dorsal view **B** Spermatheca **C** Ovipositor. Terminalia scale bars: 100 µm.

External morphology. Same as the male except abdomen, which is yellow with dorsal midline that reaches the 6^th^ tergite, 1^st^ tergite yellowish brown, 2^nd^ – 5^th^ tergites with posterior dark pigmentation which reaches and covers the exterior margin leaving a round clear area, 6^th^ tergite totally dark (Figure 10A). Wings yellow. Alar length 3.6 mm, alar width 1.38 mm. Alar indices: alar = 2.21; C = 4.32; ac =1.78; hb = 0.44; 4c = 0.51; 4v = 1.40; 5x = 1.20; M = 0.36 and prox. x = 0.42.

*Terminalia*. Spermatheca lightly pigmented, balloon-shaped, with small indents, deeply invaginated to three-quarters (Figure 10B). Ovipositor elongated, sclerotized, with 20 marginal and eight discal teeth, one long bristle and three fine hairs (Figure 10C).

Variation in paratypes (dry mounted specimen). Total length (body + wings) 4.12–5.05 mm, body length 2.49–3.27 mm. Head. Frontal length 0.31–0.39 mm, frontal index = 0.72–0.84, top to bottom width ratio = 1.16–1.55; distance of or3 to or1 = 0.08–0.15, distance of or3 to vtm = 0.11–0.16, or1-or3 ratio = 0.5–2, or2-or1 ratio = 0.10–0.46, distance of ocellar setae = 0.70–1.11 of frontal length, distance of postocellar setae = 0.64–0.88 of frontal length, vt index = 0.74–1.34; cheek index = 5.40–9.83; vibrissa index = 0.88–1.0; eye index = 1.07–1.35. Thorax. Length 0.86–1.04 mm, h index = 0.65–1.26. Transverse distance of dorsocentral setae = 1.76–2.50, dc index = 0.64–0.82. Distance between apical scutellar seta = 0.69–1.12. scut index = 1.05–1.72; sterno index = 1.20–2.23.

#### Etymology.

In Kichwa, *sachapuyu* refers to *sacha* (forest) and *puyu* (cloud). This species was found on a cloud forest habitat.

#### Distribution.

Known only from the type locality.

#### Ecology.

Unknown. The type specimen was found in the fermented banana-bait traps placed at the locality, which suggests that this species feeds and breeds in fermented fruit as do many other *Drosophila* species. This species has been reared with gelatin banana media devised by Rafael et al. 2000. The habitat is a well-preserved intermediate montane forest.

#### Relationship to other species.

The general shape of the terminalia of *Drosophilasachapuyu* sp. n. has similarities with the terminalia of *D.pichinchana* Vela & Rafael, 2004. The two smaller ventral sheets and two heavier dorsal sheets are fused by a membranous medial region. Both species have four bristles on the ventral lobe. *Drosophilasachapuyu* and *Drosophilapichinchana* differ in the general shape of the aedeagus. The aedeagus of *D.sachapuyu* is dorsally flattened.

### Drosophila misi sp. n.

Taxon classificationAnimaliaDipteraLauxaniidae

http://zoobank.org/F892199E-0A33-40A1-AE63-F0A70C124EC1

Fig. 11A–F

#### Type material.

Holotype ♂ (dissected, terminalia in microvial), Ecuador, Loja, Cajanuma, 2800 m, 4°6'58.9"S; 79°10'11.9"W, 19 Nov. 2015, A. Peñafiel col., A. Peñafiel & V. Rafael det. (QCAZ-I 3329).

#### Paratypes.

6 ♂♂ (dissected, terminalia in microvial), same data as holotype, 19 Nov. 2015, A. Peñafiel col., A. Peñafiel & V. Rafael det. (QCAZ-I 3330–3335). 1 ♂♂ (dissected, terminalia in microvial) Ecuador, Loja, Cajanuma, 2675 m, 4°6'53.7"S; 79°10'54.6"W, 19 Nov. 2015, A. Peñafiel col., A. Peñafiel & V. Rafael det. (QCAZ-I 3294).

#### Diagnosis.

Aristae with five dorsal and two ventral branches. Two prominent oral bristles. Thorax yellowish brown. Wings yellow, dM-Cu slightly clouded. Abdomen yellow with dorsal midline, 1^st^ tergite yellowish brown, 2^nd^ – 5^th^ tergites with triangular pigmentation that thins laterally, 6^th^ tergite with pigmented circle in the middle. Cerci not fused to epandrium. Aedeagus sclerotized, tube-like, apex with two dorsal sclerotized projections, dorsally with a finger-like projection covered with tiny projections. Hypandrium in shield-shape. Gonopod microtrichose bearing one long bristle. Paraphyses fused to gonopod bearing two small bristles on each side.

#### Description.

Male. Holotype external morphology: total length (body + wings) 5 mm, body length 3.40 mm. Body color yellowish brown.

*Head*. Aristae with five dorsal and two ventral branches plus terminal fork and fine hairs, pedicel and flagellomere brown. Orbital plate brown, frontal length 0.27 mm; frontal index = 0.61; top to bottom width ratio = 1.59. Distance of or3 to or1 = 0.10, distance of or3 to vtm = 0.12, or1-or3 ratio = 0.76; or2-or1 ratio = 0.39. Medial vertical seta closer to lateral vertical seta, vt index = 1.33. Ocellar triangle brown, distance of postocellar setae = 0.77 of frontal length, ocellus yellow. Frontal vitta yellowish brown. Carina prominent brown, not sulcate. Cheek index = 4.66. Two prominent oral bristles the second almost the same size as the first, vibrissa index = 1. Eyes red, eye index = 1.16.

*Thorax*. Yellowish brown, thorax length 0.85 mm, acrostichal hairs in six rows between the two anterior dorsocentral setae that decrease in number toward the rear. h index = 1.07. Transverse distance of dorsocentral setae = 2.15, dc index = 0.95. Basal scutellar setae parallel, distance between apical scutellar seta = 0.68, scut index = 1.27. Medial katepisternal seta slightly smaller than the previous seta, sterno index = 1.66. Legs yellow. Clear wings, dM-Cu slightly clouded. Alar length 3.28 mm, alar width 1.41 mm. Alar indices: alar = 2.17; C = 4.55; ac = 1.87; hb = 0.20; 4c = 0.43; 4v = 1.07; 5x = 1.12; M = 0.26 and prox. x = 0.31.

**Figure 11.**
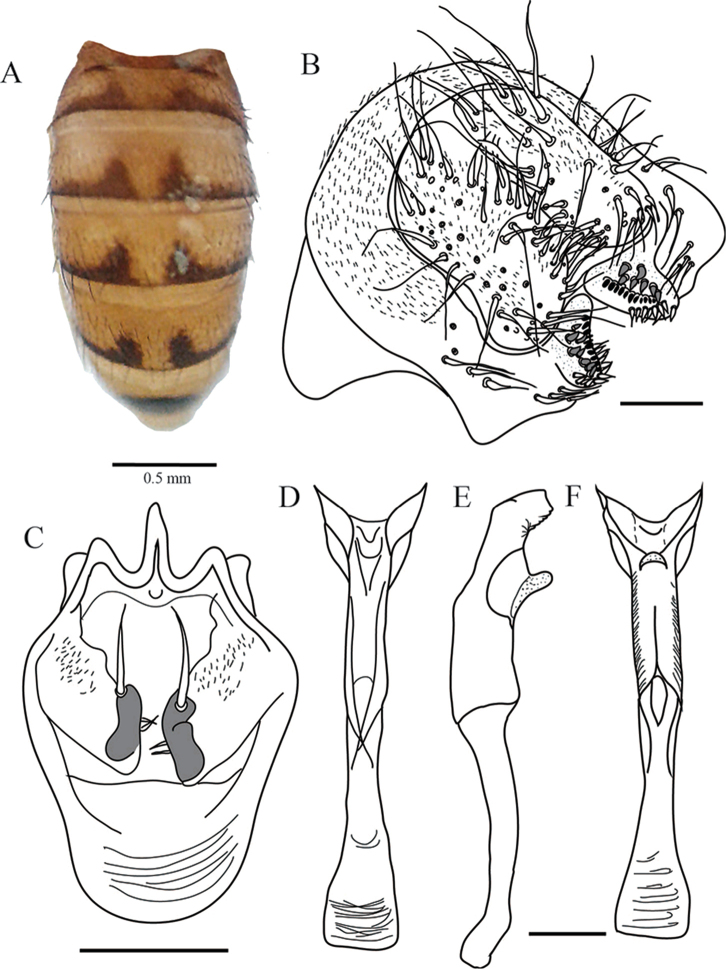
*Drosophilamisi* sp. n., abdomen and male terminalia of holotype. **A** Male abdomen, dorsal view **B** Epandrium, cerci, surstylus and decasternum **C** Hypandrium, gonopods and paraphyses, ventral view **D, E, F** Aedeagus in ventral, lateral and dorsal view, respectively. Terminalia scale bars: 100 µm.

*Abdomen*. Yellow, with dorsal midline, 1^st^ tergite yellowish brown, 2^nd^ – 5^th^ tergites with triangular pigmentation that thins laterally, 6^th^ tergite with pigmented circle in the middle (Figure 11A).

*Male terminalia.* Epandrium microtrichose, ventral lobe membranous with nine bristles. Cerci not fused to epandrium. Surstylus triangular, granular, with ten primary teeth on the right and eleven on the left, eight secondary teeth on the right and seven on the left, nine marginal bristles on the right and 12 on the left (Figure 11B). Hypandrium sclerotized, in shield-shape. Gonopod microtrichose bearing one long bristle (Figure 11C).

*Aedeagus.* Sclerotized tube-like, apex with two dorsal sclerotized projections with serrated edge. Dorsally with a finger-like projection covered in tiny projections. Ventral rod absent. Aedeagus apodeme sclerotized straight and wide (Figure 11D-F). Paraphyses strongly sclerotized fused to gonopod bearing two small bristles on each side (Figure 11C).

Variation in paratypes (dry mounted specimen). Head. Frontal length 0.26–0.31 mm, frontal index = 0.73–0.88, top to bottom width ratio = 1.51–1.68; distance of or3 to or1 = 0.07–0.09, distance of or3 to vtm = 0.10–0.14, or1-or3 ratio = 0.50–1.03, or2-or1 ratio = 0.22–0.41, distance of ocellar setae = 0.67–1.07 of frontal length, distance of postocellar setae = 0.61–0.80 of frontal length, vt index = 0.81–1.36; cheek index = 4.36–8.66; vibrissa index = 0.78–1.0; eye index = 0.95–1.36. Thorax. Length 0.84–1.03 mm, h index = 0.83–1.33. Transverse distance of dorsocentral setae = 1.30–2.31, dc index = 0.95–1.16. Distance between apical scutellar seta = 0.73–0.90. scut index = 1.23–1.27.

#### Etymology.

In Kichwa, *misi* means cat; the apex of the aedeagus resembles the ears of a cat.

#### Distribution.

*Drosophilamisi* is known from two localities (elevation range is 2675–2800 m) from Loja Province, Podocarpus National Park.

#### Ecology.

Unknown. The type specimen was found in the fermented banana-bait traps placed at the locality, which suggests that this species feeds and breeds in fermented fruit as do many other *Drosophila* species. The habitat is a well-preserved montane forest.

#### Relationship to other species.

The general shape of the male terminalia suggests that this species belongs to the *Drosophilaguarani* group

## Discussion

Nineteen species have been previously placed in the *guarani* group (Vilela and Bachli 1990; Vilela and Pereira 1993; Vela and Rafael 2004; Vela and Rafael 2005; Ratcov et al. 2017; Vaz et al. 2018). Their distribution is restricted to Central and South America (Kastritsis 1969). Collections in Loja Province of Ecuador revealed two species, *D.griseolineata* and *D.urubamba* (Rafael & Vela, 2003). *Drosophilaguarani* group has the second highest species richness after the *Drosophilatripunctata* group in Podocarpus National Park, with eleven species (Peñafiel 2016). With the five new species described in this paper, the southern Andes of Ecuador are home to almost the same number of *guarani* group species previously described.

Characteristics such as the alar pigmentation, spotted thorax and abdominal pigmentation are similar between *D.zamorana*, *D.urubamba* and *D.tucumana*. We propose the cluster formed by these three spotted-thorax species. These species clearly belongs to *guarani* group because they share two prominent oral bristles of approximately equal length, divergent anterior scutellars, cerci not fused to epandrium and shield-shaped hypandrium. Previously the *guarani* group did not include spotted-thorax species (Vilela and Val 2004).

*Drosophilamisi* sp. n. is not similar to other species in the *guarani* group. It shares the important key characters of *guarani* group. This species has a brownish body color; two prominent vibrissal setae of nearly equal size, divergent basal scutellar setae, clouded crossveins and tergites usually with interrupted, dark marginal bands (Vilela and Bächli 1990). The genitalia characteristics of *D.misi* including the cerci not being fused to the epandrium, the shield-shaped hypandrium and the general shape of the aedeagus are characteristic of species in the *guarani* group.

## Supplementary Material

XML Treatment for Drosophila zamorana

XML Treatment for Drosophila quinarensis

XML Treatment for Drosophila caxarumi

XML Treatment for Drosophila sachapuyu

XML Treatment for Drosophila misi
